# Local Dielectric Property Detection of the Interface between Nanoparticle and Polymer in Nanocomposite Dielectrics

**DOI:** 10.1038/srep38978

**Published:** 2016-12-13

**Authors:** Simin Peng, Qibin Zeng, Xiao Yang, Jun Hu, Xiaohui Qiu, Jinliang He

**Affiliations:** 1 State Key Lab of Power Systems, Department of Electrical Engineering, Tsinghua University, Beijing, 100084, China; 2 Key Laboratory of Standardization and Measurement for Nanotechnology, Chinese Academy of Sciences, National Center for Nanoscience and Technology, Beijing, 100190, China; 3 School of Chemistry and Chemical Engineering, University of Chinese Academy of Sciences, Beijing, 101408, China

## Abstract

The interface between nanoparticles and polymer matrix is considered to have an important effect on the properties of nanocomposites. In this experimental study, electrostatic force microscopy (EFM) is used to study the local dielectric property of the interface of low density polyethylene (LDPE)/TiO_2_ nanocomposites at nanometer scale. The results show that the addition of TiO_2_ nanoparticles leads to a decrease in local permittivity. We then carry out the finite element simulation and confirm that the decrease of local permittivity is related to the effect of interface. According to the results, we propose several models and validate the dielectric effect and range effect of interface. Through the analysis of DSC and solid-state NMR results, we find TiO_2_ nanoparticles can suppress the mobility of local chain segments in the interface, which influences the dipolar polarization of chain segments in the interface and eventually results in a decrease in local permittivity. It is believed the results would provide important hint to the research of the interface in future research.

Dielectric polymer nanocomposites have attracted more and more attention in recent years for their enhanced mechanical, thermal and electric properties^1^,^2^,^3^,^4^,^5^. It is believed that the interface between nanoparticles and polymer plays an important role in modifying properties of nanocomposite^6^,^7^. However, the mechanism of the enhancement has not yet been understood. The interface is the nanoscale transition region between nanoparticles and polymer matrix, because the nanoscale is far smaller than the spatial resolution of most conventional analytical measurements, direct detection of the interface is still considered to be a difficult thing. Although some models of the interface have been proposed and explained some macro experimental results^6^,^7^, the research of the interface has not made much progress.

Among electrical-characterization techniques applied in materials science, the electrostatic interaction has been widely applied in non-invasive characterization of surface charge distribution, potential profile, dielectric properties and conductivity of a variety of samples. EFM is a technique based on the accurate detection of electrostatic interaction between a scanning probe and a sample surface at the level of nanoscale^8^,^9^,^10^,^11^. With the ultra-high precision and nanometer detection resolution of EFM^12^, even very small local electrostatic interaction can be detected, which may reflect the local dielectric property of the material^13^,^14^.

## Results and Discussion

### Local Dielectric Property of LDPE/TiO_2_ nanocomposite

**Figure 1:**
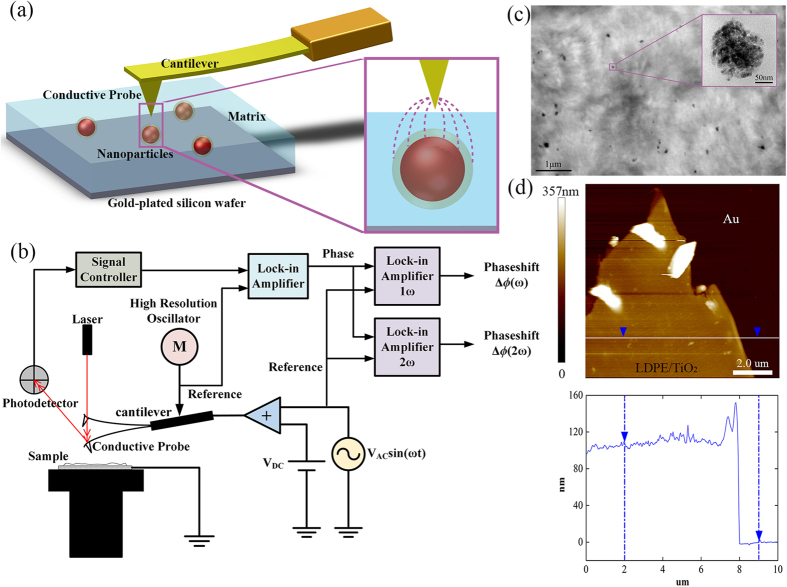
(**a**) The working schematic diagram of the EFM probe in the experiment. (**b**) The working principle of the local dielectric detection. (**c**) TEM image of LDPE/TiO_2_ nanocomposite. (**d**) The topography of test area.

The working schematic diagram of the conductive probe is shown in Fig. 1a. The working principle of the local dielectric detection with EFM is exhibited in Fig. 1b. The cantilever and the probe is driven mechanically by a piezo at its resonant frequency *f*_0_. In the first scanning, the standard tapping mode imaging is performed to obtain the topography of a scan line. In the second scanning, the topography information is used to retrace the baseline and the probe scans at a given lift height 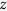 above the surface of sample^15^. An external voltage *V* = *V*_*DC*_ + *V*_*AC*_ sin(*ωt*) was applied to the probe to excite an electrostatic interaction, which will influence the vibration state of the probe, such as amplitude, frequency and phase. In this study, the 2*ω* phase shift signal Δ*ϕ*(2*ω*) is detected to avoid the impact of the work function difference between the probe and sample (Supplementary Note 2) and obtain the electrostatic force gradients^13^,^14^, which is related to the capacitance between the probe and the sample. Figure 1c shows the TEM image of LDPE/TiO_2_ nanocomposite and we find the diameter of the TiO_2_ nanoparticle is about 100 nm. Figure 1d is the topography of test area and the average thickness is about 117 nm, which indicates nanoparticles will not be stacked together in the test area.

**Figure 2:**
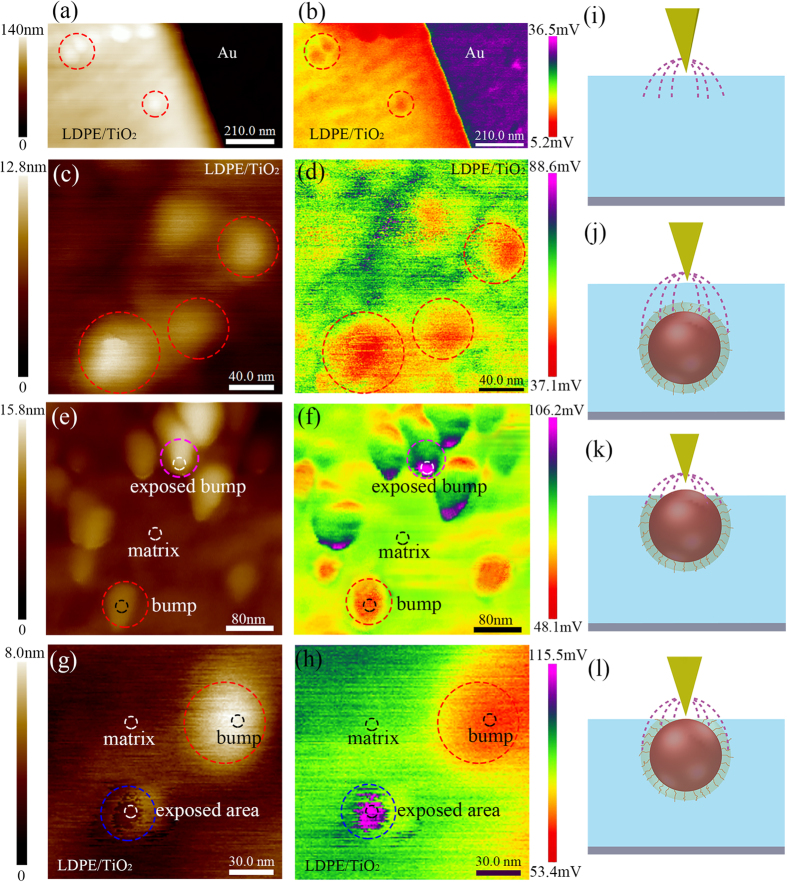
The results of local dielectric property detection. (**a**,**c**,**e** and **g**) are the topography signals, (**b**,**d**,**f** and **h**) are the |Δ*ϕ* (2*ω*)| signals amplified by the lock-in amplifier. (**i**,**j**,**k** and **l**) are four typical cases: “matrix”, “bump”, “exposed bump” and “exposed area”.

The results of local dielectric detection are shown in Fig. 2. Figure 2a and c show some bumps marked with red circles in the topography. The diameters of these bumps are consistent with TiO_2_ nanoparticles and they just correspond to the areas with small |Δ*ϕ*(2*ω*)| shown in Fig. 2b and d. As similar bumps and the change of |Δ*ϕ*(2*ω*)| are not found in pure polyethylene, it is validated that these bumps are actually nanoparticles wrapped in polyethylene matrix. At the same time, we find |Δ*ϕ*(2*ω*)| of these bump is smaller than polyethylene matrix.

Some other interesting conclusions can be drawn from Fig. 2e and f, |Δ*ϕ*(2*ω*)| signal of the special bump structure, marked with magenta circle on the top, is much stronger than that of polyethylene matrix. Because the probe scans at a lift height above the surface of sample in the second scan and the |Δ*ϕ*(2*ω*)| signal is not influenced by the topography, we suppose that the change in |Δ*ϕ*(2*ω*)| signal is caused by the change in dielectric properties of the materials. In order to confirm the speculation, we use the peak force tapping mode in the first scan and obtain the DMT modulus of the sample. After analysis (Supplementary Note 3, Fig. S4), we confirm the bump structure marked with magenta circle represents an “exposed” nanoparticle with “broken” wrapping layer, which may be caused by the process of ultrathin sections preparation. The exposed nanoparticle leads to the strong |Δ*ϕ*(2*ω*)| signal.

Figure 2g and f reveal a special case of the “exposed” nanoparticle. The area, marked with blue circle on the lower left, also corresponds to strong |Δ*ϕ*(2*ω*)| signal. However, no obvious bump structure can be observed in topography in Fig. 2g. According to the analyses above, we suppose it corresponds to the case that nanoparticle is “just” exposed and no obvious bump structure is formed. Based on the analysis above, we summarize four cases that occur in the measurement, as shown in Fig. 2i,j,k and l. For convenience, they are named “matrix” (pure polyethylene without nanoparticle), “bump” (a complete bump with a nanoparticle wrapped in), “exposed bump” (a bump structure formed by an exposed nanoparticle) and “exposed area” (a “just exposed” nanoparticle without bump structure), respectively.

The electric field induces a polarization in the sample, and the electric potential difference between them can be expressed as


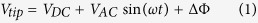


where ΔΦ represents the work function difference between the probe and the sample. Without considering the surface charge, the electrostatic force can be written as^16^


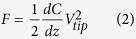


where *C* is the capacitance between the probe and the substrate. Because the van der Waals force is a kind of short-range force, while the electrostatic force is a kind of long-range force, when the probe is lifted, van der Waals force decreases rapidly^15^,^17^, the interaction force between the probe and sample is mainly the electrostatic force. The vibration state of the probe will be affected by interaction force, and the 2*ω* phase shift can be expressed as (Supplementary Note 2)


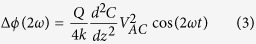


where *Q* and *k* are the quality factor and the elastic coefficient of the probe cantilever, respectively. If we keep *V*_*AC*_ constant in the scanning process, |Δ*ϕ*(2*ω*)|is directly proportional to *d*^2^*C/dz*^2^, which is closely related to the permittivity of the sample. A well-accepted model of the probe/sample capacitance can be written as^18^,^19^


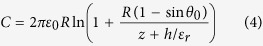


where *ε*_0_ is the permittivity of vacuum, *R* and *θ*_0_ are the tip apex radius and conical tip angle of the probe, respectively, *h* and *ε*_*r*_ are the local thickness and local relative permittivity of the sample. Because of the relationship between the 2*ω* signal and the permittivity, 2*ω* electrostatic force signal is also called dielectric force^20^.

According to the analysis, a rough calculation is conducted to calculate relative permittivity by using EFM results, we choose several points in each typical area in Fig. 2e and f and obtain topography and |Δ*ϕ*(2*ω*)| data. The calculation results of average permittivity of “matrix”, “bump” and “exposed bump” are 2.250, 1.944 and 2.546, respectively.

TiO_2_ nanoparticle has a much larger permittivity than polyethylene. If the interface is not considered, the permittivity of bump, influenced by nanoparticle wrapped in polyethylene, should be larger than that of polyethylene matrix. However, the experiment and calculation results demonstrate that the permittivity of bump is actually lower. So we guess it is the effect of interface, which leads to the decrease of the permittivity.

### Simulation of Local Dielectric Property

**Figure 3:**
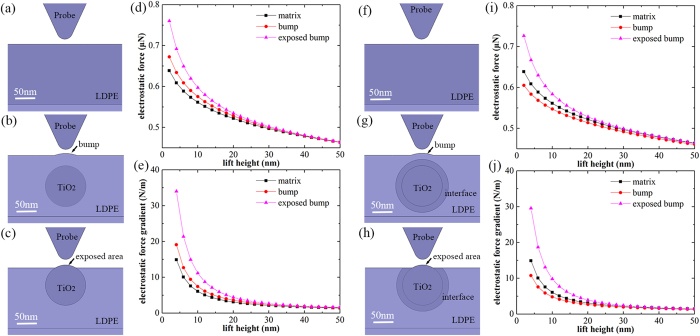
(**a**,**b** and **c**) are the local finite element models corresponding to three cases without considering the interface: “matrix”, “bump” and “exposed bump”, respectively. (**d** and **e**) are the simulation results of three models without interface. (**f**,**g** and **h**) are the local finite element models corresponding to three cases with interface: “matrix”, “bump” and “exposed bump”. (**i** and **h**) are the simulation results of three models with interface.

In order to validate the speculation, we use finite element method to simulate several situations in the experiment. At the beginning, the interface is not considered. As “exposed area” can be seen as a special case of “exposed bump” (Supplementary Note 4, Figs S6 and S7), we focus on other three cases, including “matrix”, “bump” and “exposed bump”, and design models to carry out simulation, as shown in Fig. 3a,b and c. Since the actual vibration of the probe cannot be simulated, we adopt the method of altering the lift height of probe to calculate electrostatic force in different lift height, and electrostatic force gradient can be obtained from numerical differential calculation. As the phase shift signal |Δ*ϕ*(2*ω*)| is proportional to the electrostatic force gradient signal *dF*(2*ω)dz* as previously analyzed (Supplementary Note 2), we can use the value of *dF*(2*ω)dz* to represent |Δ*ϕ*(2*ω*)| in different cases.

The results of simulation are exhibited in Fig. 3d and e. The curves of electrostatic force and electrostatic force gradient decrease with the increase of the lift height. With a fixed lift height, the electrostatic force gradient of “exposed bump” is significantly larger than that of other two cases, which is consistent with experimental results. However, the electrostatic force gradient in “bump” is larger than “matrix”, which is not consistent with experimental results.

**Figure 4:**
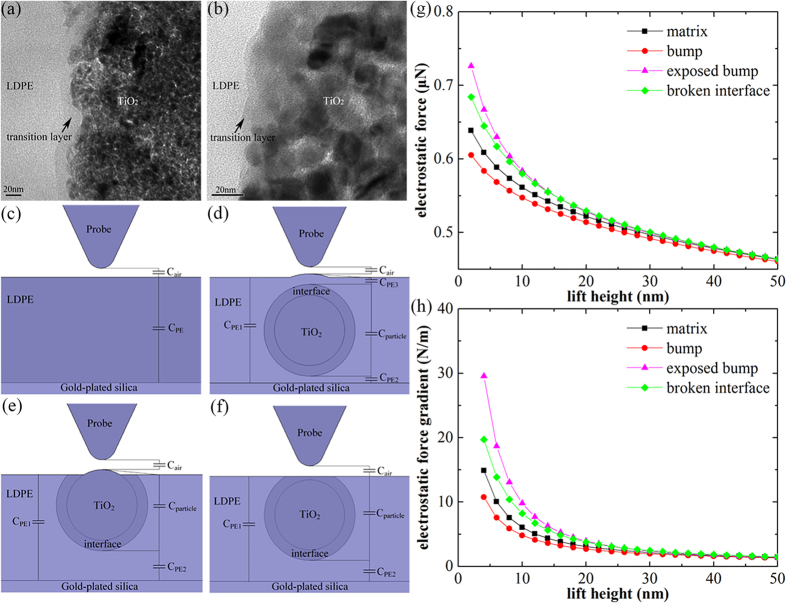
(**a** and **b**) are the transition layer between TiO_2_ nanoparticle and LDPE matrix observed by TEM. (**c**,**d**,**e** and **f**) are the capacitance structures of “matrix”, “bump”, “exposed bump” and “broken interface”. (**g** and **h**) are the simulation results of four models.

Since the simulation results are inconsistent with experiment results without considering the interface, we guess that the interface may result in a decrease of the local permittivity. We introduce a layer of interface with low permittivity outside the nanoparticle and revise the model, as shown in Fig. 3f,g and 3h. Figure 3i and j reveal that with considering the interface, the electrostatic force gradient of “matrix” is larger than that of “bump”, which is consistent with experimental results. In fact, the interface is usually regarded as a layer which has different microstructure from matrix. As a result, under some conditions, this special layer can be observed via TEM image, as shown in Fig. 4a and b, which may serve as direct evidence of the existence of the interface.

Since the |Δ*ϕ*(2*ω*)| signal is closely related to the capacitance between the probe and substrate, we analyze the capacitance structures of the models. The capacitance structures of “matrix”, “bump” and “exposed bump” are exhibited in Fig. 4c,d and e. The total capacitance measured by the probe can be decomposed into several series and parallel-connected capacitance components. It is obvious that three models have three different types of capacitance structures.

From the perspective of the capacitance structure, we can easily draw the conclusion that, as for the complete interface in “bump”, its relative permittivity and thickness will have significant influence on the dielectric response (Supplementary Note 4, Figs S8 and S9). As for the “incomplete” interface in “exposed bump”, it seems that the completeness of the interface is the key factor for the interface effect which results in the discrepancy between the experimental and simulated results of “bump” and “exposed bump”. A new model named “broken interface” has been designed to validate the speculation, as shown in Fig. 4f. The model represents a case that the interface has been partially removed while the nanoparticles has not been exposed. The completeness of the interface in “broken interface” is between “bump” and “exposed bump”, if the effect of interface is related to its completeness, the dielectric response of “broken interface” should be between “bump” and “exposed bump” as well. The simulation results, shown in Fig. 4g and h, reveal that the electrostatic force gradient of “broken interface” is between “bump” and “exposed bump”. In addition, compared with “bump”, the capacitance components of “broken interface” is more similar to “exposed bump” (both have four series and parallel-connected capacitance components), so the electrostatic force gradient of “broken interface” is also larger than “matrix”.

Considering the area around exposed area of the nanoparticle, although the interface has been partially removed during the process of ultrathin sections while the nanoparticles has not been exposed, forming a similar “broken interface” structure. Experimental results also reveal that the |Δ*ϕ*(2*ω*)| signal of the “broken interface” area is stronger than matrix (Supplementary Note 3, Fig. S4), which may serve as indirect evidence of the existence of the interface. In addition, it can also be inferred from the results that the interface effect is affected by its completeness, or the range, that is, “broken” interface may not work as well as “complete” interface. The conclusions above are summarized as the range effect of interface.

### Microscopic Mechanism of Dielectric Effect of Interface

In electromagnetics, the external electric field applied to dielectric materials will lead to polarization, which will form induced electric field and weaken external electric field. The permittivity of dielectric material is the ratio of the external electric field and actual internal electric field. That is, the permittivity is related to the polarization mechanism of the material. In the experiment, the polarization mechanism of TiO_2_ nanoparticle is ionic polarization while the polarization mechanism of polyethylene matrix and the interface is dipole polarization. Dipolar polarization is closely related to the mobility of chain segments^21^, if the chain segments are bound, the dipolar polarization will be influenced and the permittivity decreases.

**Figure 5:**
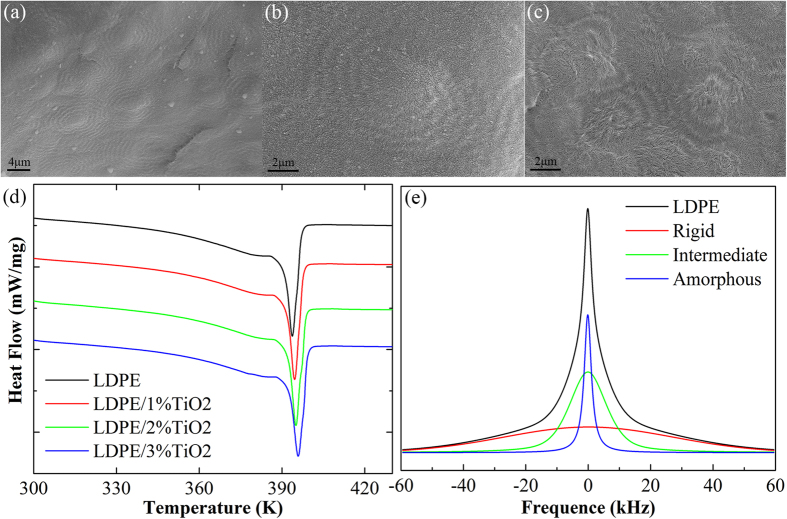
(**a** and **b**) are the SEM images of spherulites in LDPE. (**c**) is the SEM image of spherulites in LDPE/TiO_2_. (**d**) is the DSC melting curves of the LDPE and LDPE/TiO_2_ samples and (**e**) is the ^1^H wide-line solid-state NMR spectra and fitting results of LDPE.

To study the effect of TiO_2_ nanoparticles on the mobility of chain segments of LDPE, the spherulite microstructure of LDPE and nanocomposites are observed and the differential scanning calorimetry (DSC) and ^1^H solid-state nuclear magnetic resonance (SSNMR) are conducted, the results are exhibited in Fig. 5.

The morphology of the etched cross sections of pure LDPE and nanocomposites are shown in Fig. 5a,b and c, the black holes represent the original location of TiO_2_ nanoparticles, which were removed during the etching process. Figure 5a and b show that LDPE has well-defined spherulites with the average diameter of 8 μm. With the addition of TiO_2_ nanoparticles which act as the heterogeneous nucleating agents, the diameter of spherulites decreases to 3–4 μm and their number increases. It is easy to draw a conclusion that nanoparticles can affect the crystallization of the polyethylene matrix, reduce the proportion of crystalline region and increase the proportion of interphase region between crystalline region and non-crystalline region^22^.

The DSC melting curves of the LDPE and LDPE/TiO_2_ samples with different nanoparticle loading are shown in Fig. 5d. Table 1 summarizes the melting peak temperature *T*_*m*_, melting enthalpies Δ*H*_*m*_, crystallinity *X*_*c*_ and crystal thickness *L*_*c*_ of two samples, among them *L*_*c*_ is calculated by using Thomson-Gibbs equation^23^,^24^:


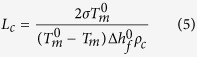


where *σ* (93 mJ/m^−2^) is the fold surface free energy, 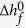 (293 J/g^−1^) is the melting enthalpies of 100% crystal-core polyethylene and 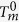 (410 K) is its equilibrium melting point, *ρ*_*c*_ (1 g/cm^3^) is the density of crystal polyethylene.

It is obvious that the melting peak temperature increases with the increase of nanoparticles, indicates that TiO_2_ nanoparticles act as the heterogeneous nucleating agents, increase crystallization temperature and accelerate the formation of crystal nucleus^25^,^26^. However, DSC crystallinity decreases with the increase of nanoparticles, which means both of them can suppress the crystallization of polyethylene. The reason for the decrease of crystallinity is likely to be the absorption of nanoparticles on polyethylene chain segments and the space steric effect of nanoparticles, meanwhile, the bound chain segments make it difficult to crystallize, as a result, large spherulites are almost impossible to be formed, just as shown in SEM images. In addition, the crystal density may decrease as the crystal thickness increases and the crystallinity decreases.

The ^1^H wide-line solid-state NMR spectra and fitting results of LDPE is exhibited in Fig. 5e. The analysis of SSNMR spectrum is based on the extent of constrained molecular motion in different polymer domains^27^,^28^. The ^1^H wide-line spectrum can be decomposed into three components by fitting the spectrum to the sum of a Gaussian function, a Lorentzian function and a combined Gaussian and Lorentzian function^28^, representing the rigid phase, the amorphous phase and the intermediate phase, respectively (Supplementary Note 5, Table S3). The phase composition can be obtained via calculating the proportion of each spectrum integration, as shown in Table 2.

NMR rigid region proportion is significantly higher than DSC crystallinity. We know that ^1^H SSNMR test is based on the mobility of molecular chain segments while DSC is based on the melting enthalpies of crystalline regions. Some chain segments near nanoparticles and structured chain segments of crystalline phase do not form lattice so they cannot contribute to the melting enthalpies of crystalline region, however, their motion are hindered with the effect of nanoparticles and structured chain segments, as a result, they behave like chain segments of crystalline phase and contribute to the proportion of rigid phase.

The result of Δ*X = X*_*rigid*_−*X*_*c*_ reveals the existence and change of bound chain segments in LDPE and its nanocomposites. In pure LDPE, Δ*X* is completely contributed by bound chain segments near crystalline phase, which are divided to non-crystalline phase in DSC experiment. After the addition of nanoparticles, Δ*X* is mainly contributed by bound chain segments around nanoparticles. The increasing of Δ*X* means that the quantity of bound chain segments increases with the increment of nanoparticles.

NMR rigid phase proportion decreases after the addition of TiO_2_ nanoparticles, compared with pure LDPE. This is mainly due to the decrease in DSC crystallinity, which contribute to the NMR rigid phase proportion. In contrast, the amorphous phase proportion increases as nanoparticles affect the crystallization process, increase the number of spherulites and reduce their size. However, with the increment of nanoparticle loading, the bound effect of nanoparticles makes more chain segments of polyethylene transfer from amorphous phase to rigid phase, so the proportion of rigid phase increases and the proportion of amorphous phase decreases.

We can also easily draw some conclusions via half-peak width Δ*υ*_*H*_ of each phase. Δ*υ*_*H*_ is inversely proportional to the apparent spin-spin relaxation time of proton *T*_2_^*^, which is an important parameters related to the molecular motion. The increase of half-peak width of rigid, intermediate and amorphous phase is found with the increment of TiO_2_ nanoparticle loading, suggesting that *T*_2_^*^ decreases and the mobility of the chain segments is more hindered^27^,^28^, which is also consistent with previous analysis.

The DSC and NMR results prove that TiO_2_ nanoparticles can suppress the mobility of local chain segments in the interface^29^,^30^,^31^, the reason may be the TiO_2_ nanoparticles form hydrogen bonds with polyethylene and coupling agent molecules, the hydrogen bond has strong attractive force, which will hinder the mobility of local chain segments^32^. As a result, the bound chain segments surrounding the nanoparticle form the interface and influence the local dipolar polarization, eventually the permittivity of the interface decreases (Supplementary Note 5, Table S4).

## Conclusions

In summary, we report an experimental measurement of the local dielectric property detection of LDPE/TiO_2_ nanocomposites. We design a series of finite element models and carry out simulation. The experimental and simulated results validate that the interface is the key factor which causes the decrease of local permittivity. The effect is named the “dielectric effect” of interface. Meanwhile, the interface needs a sufficient range to play a role, when the interface reduces, the dielectric effect is weaken as well. The conclusion above is summarized as the range effect of interface. Further researches show that interface has different microstructure. With the influence of TiO_2_ nanoparticles, the mobility of chain segments in the interface is suppressed, which influences the dipole polarization and eventually lead to the decrease in local permittivity. The experiment develops an effective method for investigating local dielectric properties of polymer nanocomposites and inspire the study on interface in the future.

## Method

### Materials Preparation

In this study, TiO_2_ nanoparticles (anatase crystal forms) were supplied by Aladdin Industrial Corporation. Low density polyethylene (LDPE) pellets were obtained from ExxonMobil, which were additive-free, melt flow 3.5 g/10 min, density 0.924 g/cm3 and melting point 124 °C. The coupling agent, (3-Aminopropyl) triethoxy-silane (code name KH550), was chosen to modify the nanoparticles to reduce agglomeration. The chemical reaction temperature was 100 °C, lasting 12 hours. With the effect of toluene, polar groups on the surface of nanoparticles, such as hydroxyl, and the coupling agents join together and generate small molecules (Supplementary Note 1, Fig. S1).

The modified TiO_2_ nanoparticles were blended with LDPE in a HAPRO torque rheometer with 60 ml Roller-cone mixer. The rotor speed was 60 rpm and the mixing time is 10 minutes. Film samples with different thickness were obtained by using compression melding at the temperature of 140 °C, lasting 10 minutes under the pressure of about 15 MPa. After that, the films were cooled to the room temperature under the same pressure. All films (Supplementary Note 1, Table S1) were annealed in the vacuum oven at 90 °C for 12 hours to eliminate the thermal history and internal stress. The FTIR spectra and XRD patterns prove that the preparation of nanocomposites was successful (Supplementary Note 1, Fig. S2). TEM image shows that TiO_2_ nanoparticles with an average diameter of about 100 nm have good dispersion in nanocomposites, as shown in Fig. 1c.

### Local Dielectric Property Detection with EFM

The local dielectric property detection is implemented by a Bruker Dimension Icon Scanning Probe Microscope. The conductive probe is a Pt/Ir coated probe.

The ultrathin sections of LDPE/TiO_2_ nanocomposite with the average thickness of about 100 nm are placed on the gold-plated silicon wafer. Several micro-areas are chosen where the local dielectric property detection is carried out (Supplementary Note 3, Table S2).

### Finite Element Simulation

The simulation models of local dielectric property detection are established by using finite element software COMSOL (Supplementary Note 4, Fig. S5). The DC (2 V) and AC (amplitude 3 V) voltage was applied to the probe, the frequency of AC voltage was set to a 1 kHz. The electrostatic force was calculated and then decomposed into DC component, ω component and 2ω component. 2ω component of the electrostatic force was selected for further calculation, just corresponding to the principle of measurement.

The model of probe, with the tip apex radius of 20 nm, the conical tip angle of 25°and the length of 12.5 μm, is built according to the parameters of an actual probe. The thick of sample is 150 nm and the diameter of TiO_2_ nanoparticle is 100 nm, which are based on the results of topography scanning and TEM, respectively. The permittivity of TiO_2_ nanoparticle and LDPE matrix is set to 80 and 2.25 respectively.

According to the experimental results, three basic models, including “matrix”, “bump” and “exposed bump” are constructed. The bump with the height of 5 nm and the diameter of about 50 nm, and the exposed bump with the height of 7 nm and the diameter of about 50 nm are set with reference to the results of topography scanning. The simulation results are shown in Fig. 3d and e.

As the simulation results of basic models are not consistent with EFM results, the interface is introduced. The thickness of interface is set to 20 nm, referring to the classical multi-core model of interface^6^, the permittivity of the interface is set to 1.6, and the simulation results are exhibited in Fig. 3i and j.

### Effect of Nanoparticles on the Aggregation Structure of Polyethylene

The samples for SEM observation were broken in liquid nitrogen in order to obtain the cross sections. The thickness of samples was about 1 mm. The cross sections were etched at room temperature for 4 hours in a 1% w/v solution of potassium permanganate in 5 parts concentrated sulphuric acid to 2 parts orthophosphoric acid to 1 part water^33^. Then the cross sections were sputter-coated with gold in order to avoid charge accumulation.

The differential scanning calorimetry (DSC) experiment is conducted to study the crystallinity of LDPE and LDPE/TiO_2_ samples under nitrogen atmosphere at a heating/cooling rate of 10 K/min between 300 and 430 K.

^1^H solid-state nuclear magnetic resonance (NMR) spectra are obtained using a Bruker^®^ AVANCE III NMR spectrometer at a proton frequency of 400.25 MHz. Results were collected for non-spinning LDPE and LDPE/TiO_2_ samples. A 1.27-μs 90° pulse with recycle delay of 5 s was used for experiments.

## Additional Information

**How to cite this article**: Peng, S. *et al*. Local Dielectric Property Detection of the Interface between Nanoparticle and Polymer in Nanocomposite Dielectrics. *Sci. Rep.* **6**, 38978; doi: 10.1038/srep38978 (2016).

**Publisher’s note:** Springer Nature remains neutral with regard to jurisdictional claims in published maps and institutional affiliations.

## Supplementary Material

Supplementary Information

## Figures and Tables

**Table 1:** Summary of DSC results of LDPE and LDPE/TiO_2_ samples.

Sample	*T*_*m*_ (K)	Δ*H*_*m*_ (J/g)	*X*_*c*_ (%)	*L*_*c*_ (nm)
LDPE	393.74	114.89	39.21	16.01
LDPE/1%TiO_2_	394.53	111.13	37.93	16.82
LDPE/2%TiO_2_	395.03	109.21	37.27	17.39
LDPE/3%TiO_2_	395.89	106.98	36.51	18.45

**Table 2:** Region proportion and line width decomposed from ^1^H solid-state NMR spectra (R for Rigid, I for Intermediate and A for Amorphous).

Sample	Phase composition (%)			Δ*X* (%)	Half-peak width (kHz)		
	R	I	A		R	I	A
LDPE	44.74	39.01	16.25	5.53	62.04	13.69	2.85
LDPE/1%TiO_2_	42.98	39.05	17.97	5.05	63.28	14.05	2.98
LDPE/2%TiO_2_	43.72	39.07	17.21	5.79	63.33	14.14	3.08
LDPE/3%TiO_2_	44.54	39.17	16.29	8.03	63.46	14.22	3.18

## References

[b1] Zhao G. . Ultralow-dielectric-constant films prepared from hollow polyimide nanoparticles possessing controllable core sizes. Chem. Mater. 21, 419–424 (2008).

[b2] Tuncer E. . Enhancement of dielectric strength in nanocompositesResearch sponsored by the Laboratory Directed Research and Development (LDRD) Program of Oak Ridge National Laboratory (ORNL), managed by UT-Battelle, LLC for the US Department of Energy under Contract No. DE-AC05-00OR22725 (D06-100). Nanotechnology. 18, 325704 (2008).

[b3] Hoyos M. . Electrical strength in ramp voltage AC tests of LDPE and its nanocomposites with silica and fibrous and laminar silicates. J. Polym. Sci. Pol. Phys. 46, 1301–1311 (2008).

[b4] Zhou Y., Hu J., Dang B. & He J. L. Titanium oxide nanoparticle increases shallow traps to suppress space charge accumulation in polypropylene dielectrics. RSC Adv. 6, 48720–48727 (2016).

[b5] Yang Y., Hu J. & He J. L. Mesoporous Nano-silica serves as the degradation inhibitor in polymer dielectrics. Sci. Rep. 6, 28749 (2016). 27338622 10.1038/srep28749 PMC4919646

[b6] Tanaka T., Kozako M., Fuse N. & Ohki Y. Proposal of a multi-core model for polymer nanocomposite dielectrics. IEEE. T. Dielect. El. In. 12, 669–681 (2005).

[b7] Lewis T. J. Interfaces are the dominant feature of dielectrics at the nanometric level. IEEE. T. Dielect. El. In. 11, 739–753 (2004).

[b8] Ju B. F., Ju Y. & Saka M. Quantitative measurement of submicrometre electrical conductivity. Phys. D: Appl. Phys. 40, 7467 (2007).

[b9] Krauss T. D. & Brus L. E. Charge, polarizability, and photoionization of single semiconductor nanocrystals. Phys. Rev. Lett. 83, 4840 (1999).

[b10] Nakagiri N. . Application of scanning capacitance microscopy to semiconductor devices. Nanotechnology. 8, A32 (1997).

[b11] Coffey D. C. & Ginger D. S. Time-resolved electrostatic force microscopy of polymer solar cells. Nat. Mater. 5, 735–740 (2006). 16906141 10.1038/nmat1712

[b12] Colchero J., Gil A. & Baró A. M. Resolution enhancement and improved data interpretation in electrostatic force microscopy. Phys. Rev. B. 64, 245403 (2001).

[b13] Labardi M. . Local dielectric spectroscopy of nanocomposite materials interfaces. J. Vac. Sci. Technol. B. 28, C4D11–C4D17 (2010).

[b14] Fumagalli L. . Nanoscale capacitance imaging with attofarad resolution using ac current sensing atomic force microscopy. Nanotechnology. 17, 4581 (2006). 21727580 10.1088/0957-4484/17/18/009

[b15] Zhang D. . Lateral Resolution and Signal to Noise Ratio in Electrostatic Force Detection Based on Scanning Probe Microscopy. Chinese. Phys. Lett. 29, 070703 (2012).

[b16] Lu W., Wang D. & Chen L. Near-static dielectric polarization of individual carbon nanotubes. Nano. Lett. 7, 2729–2733 (2007). 17705550 10.1021/nl071208m

[b17] Saint Jean M., Hudlet S., Guthmann C. & Berger J. Van der Waals and capacitive forces in atomic force microscopies. J. Appl. Phys. 86, 5245–5248 (1999).

[b18] Fumagalli L., Ferrari G., Sampietro M. & Gomila G. Dielectric-constant measurement of thin insulating films at low frequency by nanoscale capacitance microscopy. Appl. Phys. Lett. 91, 243110 (2007).

[b19] Hudlet S., Saint Jean M., Guthmann C. & Berger J. Evaluation of the capacitive force between an atomic force microscopy tip and a metallic surface. Eur. Phys. J. B. 2, 5–10 (1998).

[b20] Zhang J., Lu W., Li Y., Cai J. & Chen L. Dielectric Force Microscopy: Imaging Charge Carriers in Nanomaterials without Electrical Contacts. Accounts. Chem. Res. 48, 1788–1796 (2015). 10.1021/acs.accounts.5b00046 26061707

[b21] Stockmayer W. H. Dielectric dispersion in solutions of flexible polymers. Pure Appl. Chem. 15, 539–554 (1967).

[b22] Huang X., Jiang P. & Yin Y. Nanoparticle surface modification induced space charge suppression in linear low density polyethylene. Appl. Phys. Lett. 95, 242905 (2009).

[b23] Mattozzi A., Neway B., Hedenqvist M. S. & Gedde U. W. Morphological interpretation of n-hexane diffusion in polyethylene. Polymer 46, 929–938 (2005).

[b24] Hedenqvist M., Angelstok A., Edsberg L., Larsson P. T. & Gedde U. W. Diffusion of small-molecule penetrants in polyethylene: free volume and morphology. Polymer 37, 2887–2902 (1996).

[b25] Xu J., Zhao Y., Wang Q. & Fan Z. Isothermal crystallization of intercalated and exfoliated polyethylene/montmorillonite nanocomposites prepared by *in situ* polymerization. Polymer. 46, 11978–11985 (2005).

[b26] Xu J., Wang Q. & Fan Z. Non-isothermal crystallization kinetics of exfoliated and intercalated polyethylene/montmorillonite nanocomposites prepared by *in situ* polymerization. Eur. Polym. J. 41, 3011–3017 (2005).

[b27] Massiot D. . Modelling one‐and two‐dimensional solid‐state NMR spectra. Magn. Reson. Chem. 40, 70–76 (2002).

[b28] Chen T., Yang H. & Li W. Phase structure and mechanical properties of disentangled ultra-high molecular weight polyethylene/polyhedral oligomeric silsesquioxane nanocomposites in a solid state. J. Polym. Res. 22, 1–8 (2015).

[b29] Ash B. J. . Mechanical properties of Al2O3/polymethylmethacrylate nanocomposites. Polym. Composite. 23, 1014–1025 (2002).

[b30] Zhu L. . Confinement-induced deviation of chain mobility and glass transition temperature for polystyrene/Au nanoparticles. Macromolecules. 46, 2292–2297 (2013).

[b31] Cousin P. & Smith P. Dynamic mechanical properties of sulfonated polystyrene/alumina composites. J. Polym. Sci. Pol. Phys. 32, 459–468 (1994).

[b32] Peng J. . An Electrostatic Force Microscopy Investigation of the Dynamic Properties of Microscopic Interface in Nanocomposites. Acta. Phys-Chim. Sin. 29, 1603–1608 (in Chinese) (2013).

[b33] Huang X., Xie L., Jiang P., Wang G. & Yin Y. Morphology studies and ac electrical property of low density polyethylene/octavinyl polyhedral oligomeric silsesquioxane composite dielectrics. Eur. Polym. J. 45, 2172–2183 (2009).

