# Supplementary material for: Local Dielectric Property Detection of the Interface between Nanoparticle and Polymer in Nanocomposite Dielectrics
Source: Sci Rep. 2016 Dec 13;6:38978. doi: 10.1038/srep38978 (PMC5154196; doi:10.1038/srep38978)
Supplement: Supplementary Information [file srep38978-s1.doc]

# Supplementary Information

**Local Dielectric Property Detection of the Interface between Nanoparticle and Polymer in Nanocomposite Dielectrics**

Simin Peng ^1^, Qibin Zeng ^2, 3^, Xiao Yang ^1^, Jun Hu ^1^, Xiaohui Qiu ^2^ and Jinliang He^*, 1^

^1^State Key Lab of Power Systems, Department of Electrical Engineering, Tsinghua University, Beijing, 100084, China

^2^Key Laboratory of Standardization and Measurement for Nanotechnology, Chinese Academy of Sciences, National Center for Nanoscience and Technology, Beijing, 100190, China

^3^School of Chemistry and Chemical Engineering, University of Chinese Academy of Sciences, Beijing, 101408, China

Correspondence and requests for materials should be addressed to J.L.H. (email: [hejl@tsinghua.edu.cn](mailto:hejl@tsinghua.edu.cn).)

**Supplementary Note 1: Preparation and Characterization of LDPE/TiO_2_ nanocomposite.**


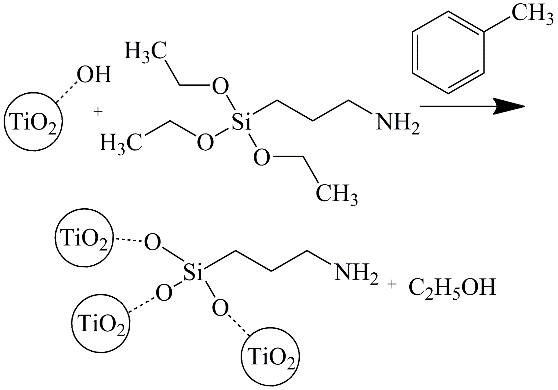


**Figure S1** The chemical reaction principle of surface modification.

The coupling agent, (3-Aminopropyl) triethoxy-silane (code name KH550), was chosen to modify the nanoparticles to reduce agglomeration. The chemical reaction temperature was 100 °C, lasting 12 hours. With the effect of toluene, polar groups on the surface of nanoparticles, such as hydroxyl, and the coupling agents join together and generate small molecules.

The nanoparticles were dispersed in the polyethylene by using a melt blending method. The composition of the LDPE/MgO nanocomposites was shown in Table S1.

**Table S1** Sample Notation and composition.

| Sample | Modified TiO_2_ (wt %) | LLDPE (wt %) |
| --- | --- | --- |
| LDPE | 0 | 100 |
| LDPE/1%TiO_2_ | 1.0 | 99.0 |
| LDPE/2% TiO_2_ | 2.0 | 98.0 |
| LDPE/3% TiO_2_ | 3.0 | 97.0 |


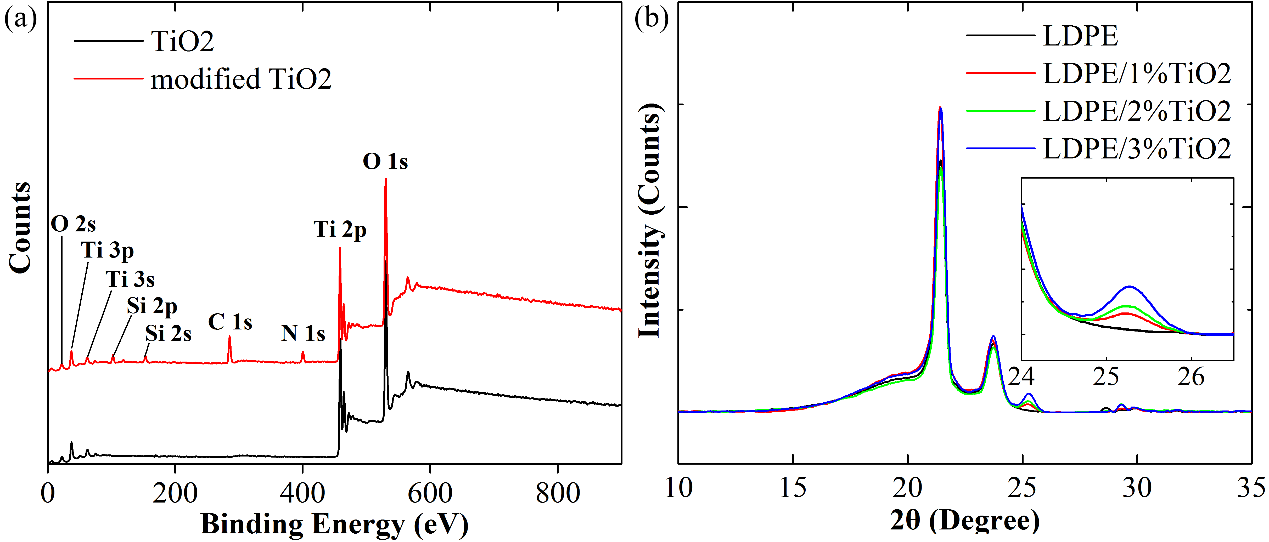


**Figure S2** (a) The XPS spectra of TiO_2_ with and without modification. (b) The XRD patterns of LDPE and LDPE/TiO2 nanocomposites.

The X-ray photoelectron spectra (XPS) of the samples were recorded using a Kratos Analytical Spectrometer with a monochromated Al Kα source to investigate the detailed chemical structures of TiO_2_ after the functionalization with KH550. As shown in Figure S2a, compared with the XPS spectrum of TiO_2_, the appearance of the Si 2p peak at about 100 eV, the Si 2s peak at about 150 eV, C 1s peak at about 285 eV and N 1s peak at about 400 eV in modified TiO_2_^1^ confirm the successful modification of KH550. These results prove that the surface modification of nanoparticles were successful.

The XRD patterns were obtained at room temperature by a Siemens 08DISCOVER High-resolution X-ray diffractometer, using Cu Kα radiation (λ = 1.5406 Å) in the range of 2θ from 5º to 140º, and a scanning rate of 0.02 s^-1^. XRD patterns of LDPE and LDPE/TiO_2_ nanocomposites are shown in Figure S2b, two sharp peaks at 21.52° and 23.9°, corresponding to (110) and (200) planes respectively were characteristic for LDPE phase^2^. After being dispersed of the nanoparticles in LDPE, new typical peak at 25.26° appears, representing (110) plane of TiO_2_ nanoparticles in anatase phase^3^. These results prove that the dispersion of TiO_2_ nanoparticles were successful.

**Supplementary Note 2: Mechanism of Local Dielectric Property Detection**

In the first scanning, the standard tapping mode imaging is performed to obtain the topography of a scan line. In the second scanning, the topography information is used to retrace the baseline and the probe scans at a given lift height
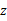
 above the surface of sample^4^. An external voltage $V=V_{DC}+V_{AC}\sin\left(\omega t\right)$ was applied to the probe and the electric field between the probe and the sample induces a polarization in the sample, and the electric potential difference between them can be expressed as

$V_{tip}=V_{DC}+V_{AC}\sin\left(\omega t\right)+\Delta\Phi$ (1)

where $\Delta\Phi$ represents the work function difference between the probe and the sample. Without considering the surface charge, the electrostatic force can be written as^5^

$F=\frac{1}{2}\frac{dC}{dz}V_{tip}^{2}$ (2)

where $C$ is the capacitance between the probe/cantilever and the sample. Because van der Waals force is a kind of short-range force, while electrostatic force is a kind of long-range force, when the probe is lifted, van der Waals force decreases rapidly^4^, the interaction force between the probe and sample is mainly the electrostatic force.

Due to the impact of interaction force, the vibration state of the probe such as amplitude, frequency and phase changes and EFM can detect minor changes of these parameters. In the phase modulation mode, the vibration can be described as a forced Lorentz oscillator model^6^:

(3)

where$m$, $Q$ and $k$ are the mass, quality factor and elastic coefficient of the probe cantilever, respectively, $x$and $\Omega_{0}$ are the vibration displacement and resonance angular frequency of the probe cantilever, respectively, and$\Omega_{0}=\sqrt{k/m}$, $F_{d}$ and $\Omega$ are the amplitude and angular frequency of driving force applied on the probe cantilever, $F_{ps}$is the interaction force between the probe and sample.

Without out considering$F_{ps}$, the vibration displacement of the probe cantilever can be solved as

(4)

The steady-state solution is

(5)

$A=\frac{F_{d}}{m\sqrt{\left(\frac{\Omega_{0}\Omega}{Q}\right)^{2}+\left(\Omega^{2}-\Omega_{0}^{2}\right)}}$ (6)

(7)

Without out considering$F_{ps}$, the probe is controlled to vibrate near the resonance frequency, $\Omega=\Omega_{0}$, , which means the phase difference between probe vibration and driving force is$\frac{\pi}{2}$. It is widely recognized that $F_{ps}$ will change the elastic coefficient^2^, that is, $k_{eff}=k+\sigma$ , and $\sigma=\frac{dF_{ps}}{dz}$is the gradient of$F_{ps}$. So the phase difference between probe vibration and driving force is

(8)

So the phase shift cause by the interaction force $F_{ps}$ can be expressed as

(9)

Without considering van der Waals force. The interaction force is equal to the electrostatic force, that is,

(10)

The signal can be separated to 1*ω* signal and 2*ω* signal with a lock-in amplifier, which are given by

(11)

(12)

If we keep $V_{AC}$ constant in the scanning process, the amplitude of, that is, is directly proportional to$d^{2}C/dz^{2}$, which is closely related to the permittivity of the sample.

Usually, the $C$ contribution from the cantilever can be neglected because the distance between the cantilever and the sample (>10μm) is much greater than the amplitude of variation. As a result, we only need to consider the contribution from the probe. However, the shape of the real probe is complex, so researchers usually use a conical probe model. A well-accepted model of the probe/sample capacitance can be written as^7, 8^

$C=2\pi\varepsilon_{0}R\ln\left(1+\frac{R\left(1-\sin\theta_{0}\right)}{z+h/\varepsilon_{r}}\right)$ (13)

$\frac{d^{2}C}{dz^{2}}=2\pi\varepsilon_{0}R^{2}\left(1-\sin\theta_{0}\right)\frac{2\left(z+h/\varepsilon_{r}\right)+R\left(1-\sin\theta_{0}\right)}{\left(\left(z+h/\varepsilon_{r}\right)^{2}+\left(z+h/\varepsilon_{r}\right)R\left(1-\sin\theta_{0}\right)\right)^{2}}$ (14)

where $\varepsilon_{0}$ is the permittivity of vacuum, $R$and $\theta_{0}$ are the tip apex radius and conical tip angle of the probe, respectively, $h$and $\varepsilon_{r}$ are the local thickness and local relative permittivity of the sample. Therefore, we can obtain the relationship between $d^{2}C/dz^{2}$and$\varepsilon_{r}$by using a set of typical values, and the result is shown in Figure S3.


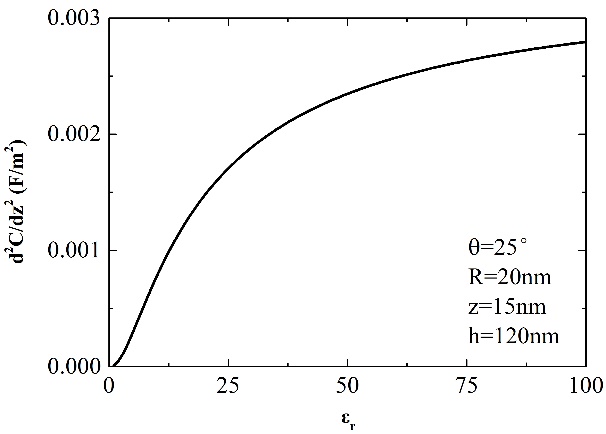


**Figure S3** The relationship between d^2^*C*/d*z*^2^ and local relative permittivity.

As shown in Figure S3, there is a positive correlation between $d^{2}C/dz^{2}$and$\varepsilon_{r}$, while is directly proportional to$d^{2}C/dz^{2}$, so we can determine the value of local relatively permittivity by comparing the amplitude of 2*ω* phase shift signal.

**Supplementary Note 3: Local Dielectric Property Detection**

The parameters of the local dielectric detection is exhibited in Table S2.

**Table S2 Parameters of the local dielectric detection**

| Parameters | value |
| --- | --- |
| Quality factor (*Q*) | 181 |
| Elastic coefficient (*k*) | 3 N/m |
| DC voltage (*V*_DC_) | 2 V |
| AC voltage (*V*_AC_) | 3 V |
| Lift height (*z*) | 15 nm |
| Amplification of Lock-in amplifier | 20 |

In order to make the conclusion more convincing, we use the peak force tapping mode to replace the tapping mode in the first scan, which is a unique working mode where the cantilever is oscillated well below resonance resulting in a continuous series of force-distance curves. On the one hand, the force between the sample and the probe is smaller and the topography signal should not be influenced by the probe. On the other hand, with the unique peak force QNM workspace of the microscope, we can get the DMT modulus of the sample, which is a more favorable evidence to explain our conclusions. The results are shown in Figure S4.


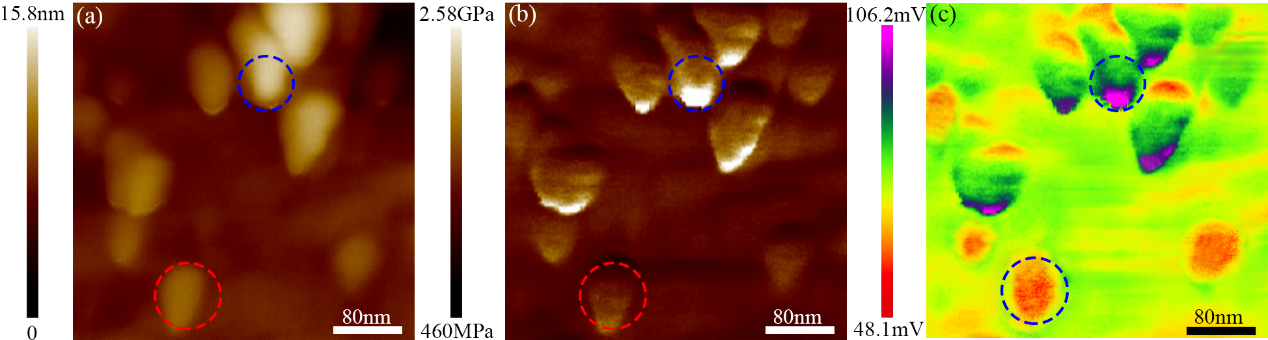


**Figure S4** The experimental results of local dielectric property detection. (a) is the topography signal, (b) is the DMT modulus signal and (c) is the |Δ*ϕ*(2*ω*)| signal.

It is obvious that area marked with blue circle has a much larger modulus than matrix, the modulus of the area is about 2.58GPa while that of the matrix is about 500 MPa, so it may not be the same material as the matrix. Considering that it also has stronger |Δ*ϕ*(2*ω*)| signal than the matrix, so we suppose it is the effect of the TiO_2_ nanoparticle and the “exposed area” does exist. The area marked with red circle, whose modulus is almost the same as that of the matrix, is considered to be the “bump”, with the effect of the interface, the |Δ*ϕ*(2*ω*)| signal is smaller than matrix.

**Supplementary Note 4: Finite Element Simulation of Dielectric Effect of Interface**

Three basic cases, including “matrix”, “bump” and “exposed bump”, are selected to build finite element models by using finite element software COMSOL. The overall simulation models are exhibited in Figure S5a. The bump with the height of 5 nm and the diameter of about 50 nm, and the exposed bump with the height of 7 nm and the diameter of about 50 nm are set with reference to the results of topography scanning, as shown in Figures S5b and S5c.


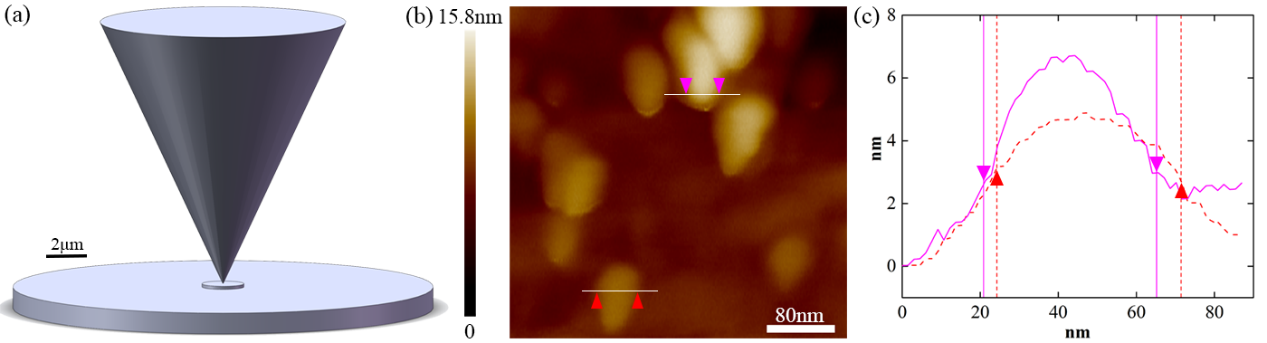


**Figure S5** (a) The overall FEM model of local dielectric property detection. (b) and (c) are the topography data sources of “bump” and “exposed bump” used for modeling.

As for “exposed area”, it can be regarded as the special case of “exposed bump” as the only difference between them is the exposed height of nanoparticle, the exposed height in “exposed area” is very small while in “exposed bump”, it is so significant that forms a bump structure. By analyzing the capacitance structure of the two models, it is found that both of them have similar capacitance structure, as shown in Figure S6.


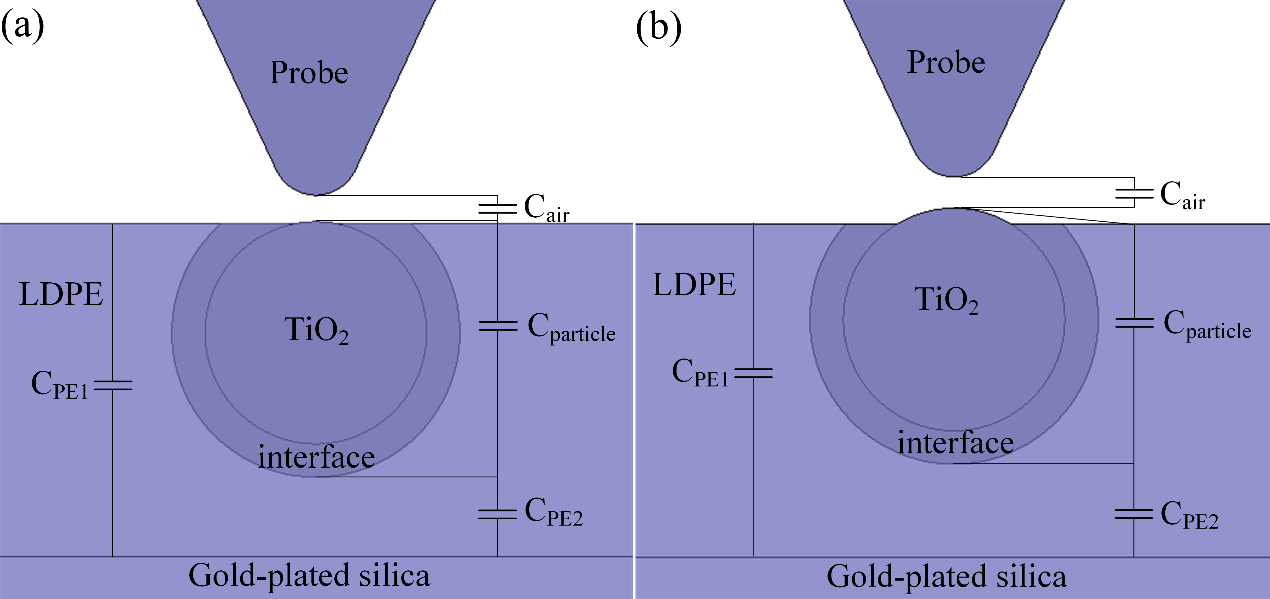


**Figure S6** The capacitance structures of the (a) “exposed area” model and (b) “exposed bump” model.

The total capacitance measured by the probe can be decomposed into four series and parallel-connected capacitance components, which means both of two models have similar dielectric response.

$\frac{1}{C}=\frac{1}{C_{air}}+\frac{1}{C_{PE1}+\frac{1}{\frac{1}{C_{particle}}+\frac{1}{C_{PE2}}}}$ (15)

It can be predictable that the |Δ*ϕ*(2*ω*)| signal of “exposed bump” is very close to that of “exposed area” and the simulation results also validate the conclusion, as shown in Figure S7. The simulation results also reveal that within a certain range, the exposed height has no significant effect on the electrostatic force gradient.


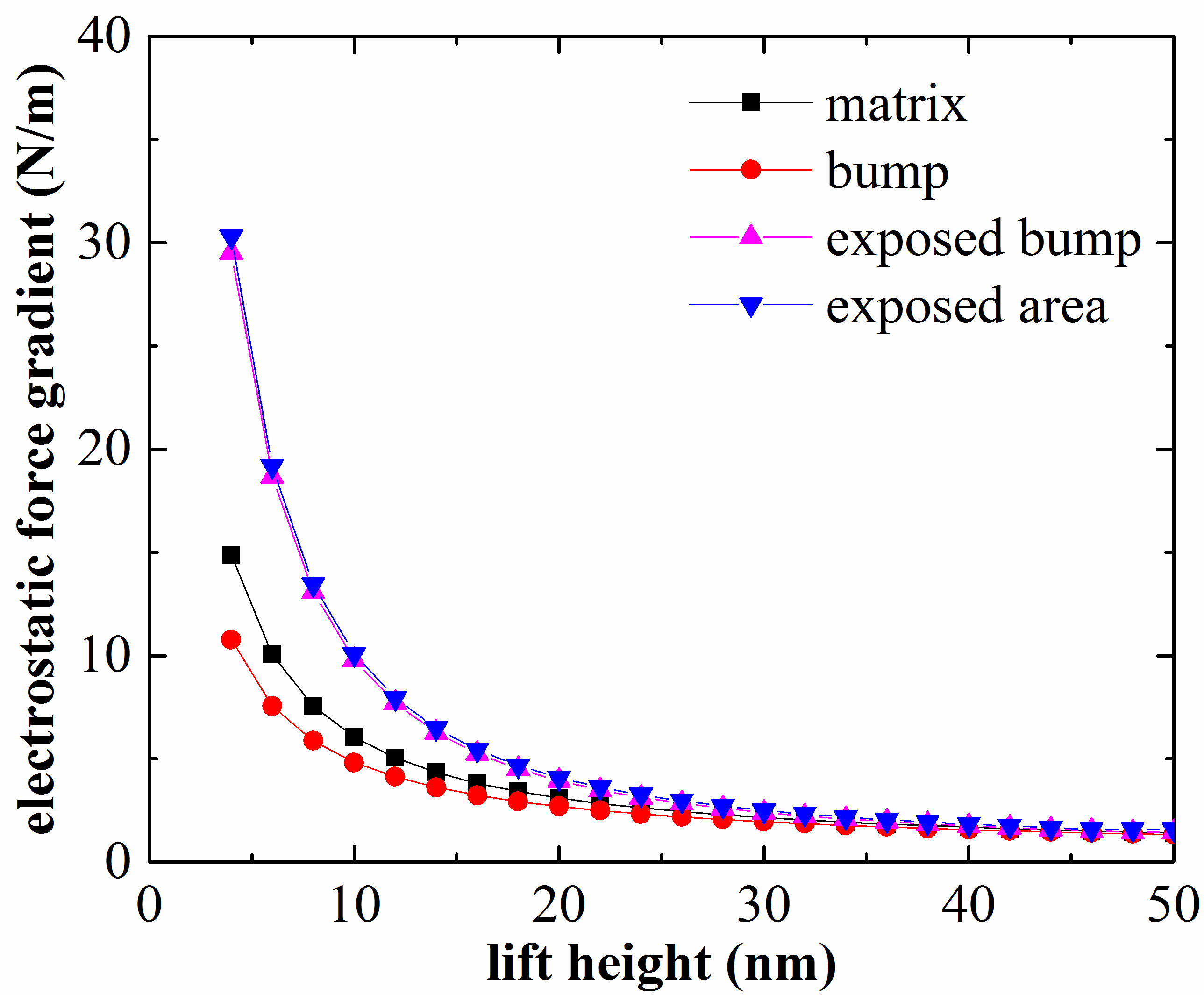


**Figure S7** The simulation results of four models with interface.


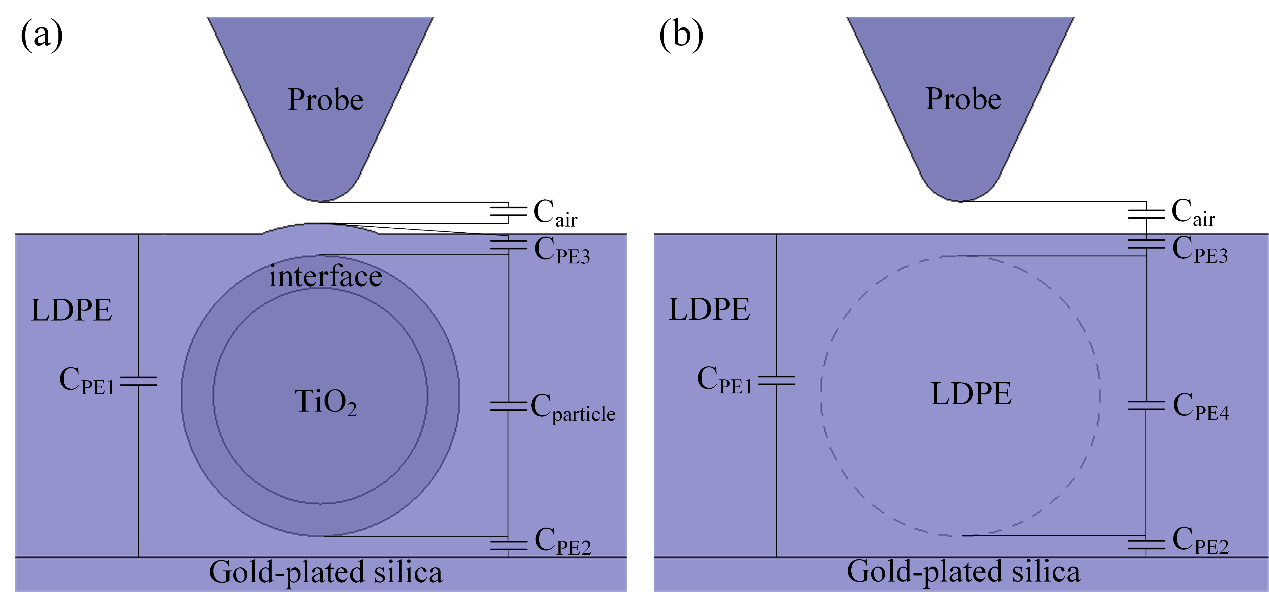


**Figure S8** The capacitance structure of the (a) “bump” model and (b) “matrix” model.

The relationship between the effect of interface and its parameters has been discussed from the perspective of the capacitance structure. As shown in Figure S8a, in the model of “bump”, the total capacitance measured by the probe can be decomposed into five series and parallel-connected capacitance components.

$\frac{1}{C_{bump}}=\frac{1}{C_{air}}+\frac{1}{C_{PE1}+\frac{1}{\frac{1}{C_{PE2}}+\frac{1}{C_{particle}}+\frac{1}{C_{PE3}}}}$ (16)

As for “matrix” model, we can assume that there is a “LDPE” nanoparticle with the same size of TiO_2_ nanoparticle (interface included), then “matrix” has similar capacitance structure with “bump” and the total capacitance can also be decomposed into five series and parallel-connected capacitance components.

$\frac{1}{C_{matrix}}=\frac{1}{C_{air}}+\frac{1}{C_{PE1}+\frac{1}{\frac{1}{C_{PE2}}+\frac{1}{C_{PE4}}+\frac{1}{C_{PE3}}}}$ (17)

Ignoring subtle differences of parameters caused by the bump structure, when the interface is not considered, it is obvious that $C_{particle}>C_{PE4}$, so the dielectric response of “bump” will be stronger than that of “matrix”. Only when the interface with low relative permittivity exists, $C_{PE4}$may be greater than $C_{particle}$ and the dielectric response of “matrix” will be stronger than that of “bump”.

Based on the analysis above, we can also draw the conclusion that the parameters of the interface will have significant influence on the total capacitance. The effect of the interface with different thickness and relative permittivity on the simulation results was studied, as shown in Figure S9.


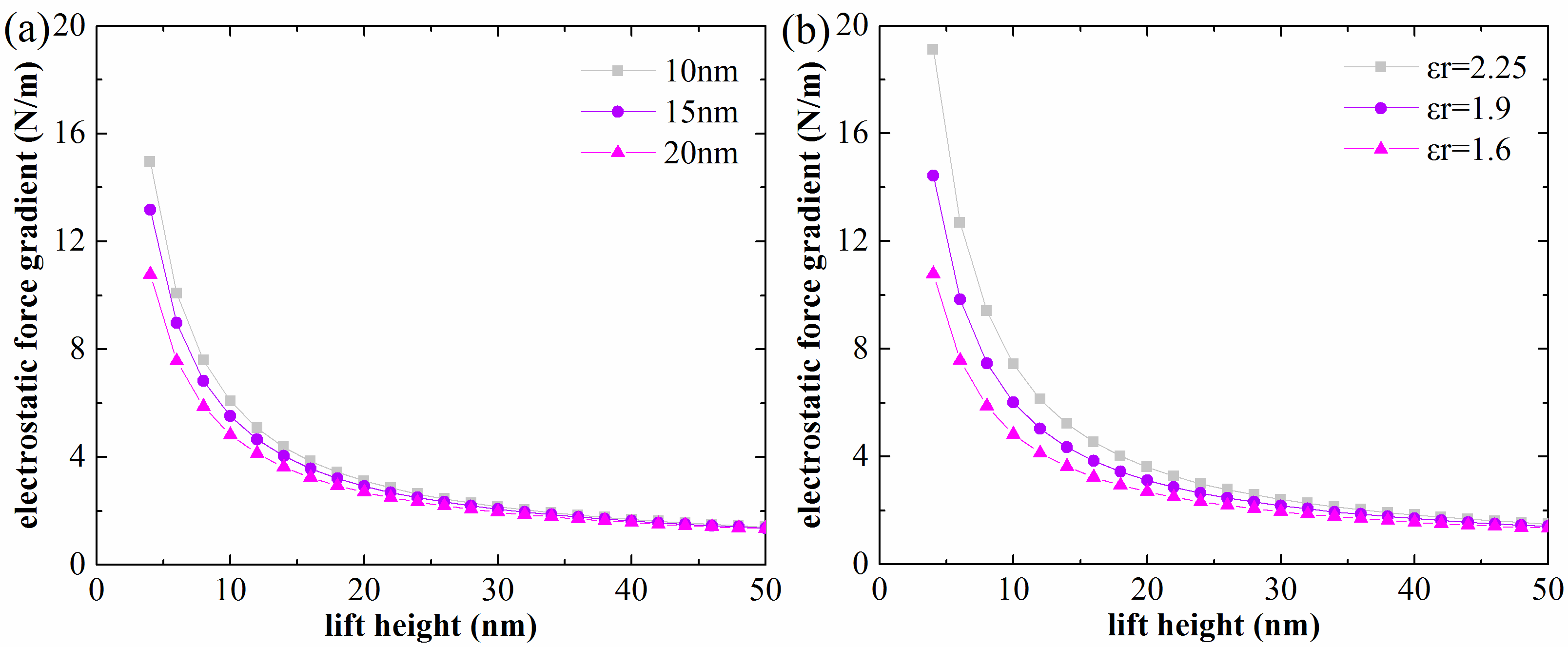


**Figure S9** The simulation results of “bump with interface” in the case of (a) different thickness of the interface with the relative permittivity of 1.6 and (b) different relative permittivity of the interface with the thickness of 20 nm.

When the parameters of the interface are in the appropriate range (for example, thickness=20nm and ε_r_=1.6), the electrostatic force gradient of the “bump” will be significantly smaller than that of the “matrix”. According to the simulation results, it is not difficult for us to draw the conclusion that interface with certain thickness and low relative permittivity does have the ability to decrease the local relative permittivity.

**Supplementary Note 5: Microscopic Mechanism of Dielectric Effect of Interface**

We use a free software DMFIT (<http://nmr.cemhti.cnrs-orleans.fr/dmfit/>) to decompose the ^1^H wide-line spectrum. The ^1^H wide-line spectrum is decomposed into three components by fitting the spectrum to the sum of a Gaussian function, a Lorentzian function and a combined Gaussian and Lorentzian function, corresponding to the rigid phase, intermediate and amorphous phase, respectively. The accuracy of decomposition can be controlled by the software. The decomposition result of LDPE spectra given by the software is listed in Table S3.

**Table S3** The decomposition result of LDPE spectra

| Phase | Parameter | Value (Hz) | Error (Hz) | Deviation |
| --- | --- | --- | --- | --- |
| Rigid | Position | 48.69652 | ± 1.77005 |  |
|  | Amplitude | 3.73973 | ± 0.00263 | 0.07% |
|  | Width | 62047.51323 | ± 25.35405 | 0.04% |
| Intermediate | Position | 45.20689 | ± 1.50873 |  |
|  | Amplitude | 12.28924 | ± 0.00924 | 0.08% |
|  | Width | 13678.17212 | ± 7.86193 | 0.06% |
| Amorphous | Position | -21.93699 | ± 0.41679 |  |
|  | Amplitude | 20.73229 | ± 0.00954 | 0.05% |
|  | Width | 2851.43296 | ± 2.26739 | 0.08% |

The phase composition is obtained by calculating the integration of each curve, which is related with the amplitude and width of the curve. Since the deviations of amplitude and width are less than 0.1%, we suppose the accuracy is enough to support our conclusions.

In addition, we have prepared new LDPE/TiO_2_ nanocomposites by using the TiO_2_ nanoparticles with different dimensions and obtained the ^1^H wide-line solid-state NMR spectra. The results of three samples with different nanoparticle diameter and same nanoparticle loading (2%) are shown in Table R1. It is found that with the increase of nanoparticle diameter, the rigid phase proportion decreases and the amorphous phase proportion increases. The half-peak width is inversely proportional to *T*_2_^*^, which is an important parameters related to the molecular motion. The decrease of half-peak width in rigid, intermediate and amorphous phases is found with the increase of nanoparticle diameter, suggesting that *T*_2_^*^ increases and the bound effect on the chain segments is weakened. We suppose that the dimension of nanoparticle has influence on the bound effect. We know the bound effect is affected by the surface state of the nanoparticle, including the groups grafted on the surface and the size of surface area. After the same surface modification process, the nanoparticle with smaller dimension will have higher specific surface area, which makes the bound effect stronger.

**Table S4 Region proportion and line width decomposed from ^1^H solid-state NMR spectra (R for Rigid, I for Intermediate and A for Amorphous)**

| Sample | Phase composition (%) | | | Half-peak width (kHz) | | |
| --- | --- | --- | --- | --- | --- | --- |
|  | R | I | A | R | I | A |
| LDPE | 44.74 | 39.01 | 16.25 | 62.04 | 13.69 | 2.85 |
| LDPE/40nm-TiO_2_ | 43.94 | 38.91 | 17.15 | 63.93 | 14.35 | 3.12 |
| LDPE/100nm-TiO_2_ | 43.72 | 39.07 | 17.21 | 63.33 | 14.14 | 3.08 |
| LDPE/200nm-TiO_2_ | 41.72 | 39.09 | 19.19 | 62.24 | 14.00 | 2.95 |

**Supplementary Reference**

1. H. Chen, M. Zheng, H. Sun, and Q. Jia, *Mat. Sci. Eng.*, 2007, **445**, 725
2. V. G. Nguyen, H. Thai, D. H. Mai, H. T. Tran, and M. T. Vu, *Compos. Part. B-Eng.*, 2013, **45**, 1192
3. D. Sarmah, J. R. Deka, S. Bhattacharyya, and N. S. Bhattacharyya, *J. Electron. Mater.*, 2010, **39**, 2359
4. D. Zhang, X. Wang, R. Wang, S. Wang, Z. Cheng and X. Qiu, *Chinese. Phys. Lett.*, 2012, **29**, 070703.
5. W. Lu, D. Wang and L. Chen, *Nano. Lett.*, 2007, **7**, 2729.
6. R. Garcıa and R. Perez, *Surf. Sci. Rep*., 2002, **47**, 197.
7. L. Fumagalli, G. Ferrari, M. Sampietro and G. Gomila, *Appl. Phys. Lett.*, 2007, **91**, 243110.
8. S. Hudlet, M. Saint Jean, C. Guthmann and J. Berger, *Eur. Phys. J. B.*, 1998, **2**, 5.
